# Training approach-avoidance of smiling faces affects emotional vulnerability in socially anxious individuals

**DOI:** 10.3389/fnhum.2013.00481

**Published:** 2013-08-20

**Authors:** Mike Rinck, Sibel Telli, Isabel L. Kampmann, Marcella L. Woud, Merel Kerstholt, Sarai te Velthuis, Matthias Wittkowski, Eni S. Becker

**Affiliations:** Behavioural Science Institute, Radboud University NijmegenNetherlands

**Keywords:** social anxiety, AAT training, face turn AAT, approach-avoidance modification

## Abstract

Previous research revealed an automatic behavioral bias in high socially anxious individuals (HSAs): although their explicit evaluations of smiling faces are positive, they show automatic avoidance of these faces. This is reflected by faster pushing than pulling of smiling faces in an Approach-Avoidance Task (AAT; Heuer et al., 2007). The current study addressed the causal role of this avoidance bias for social anxiety. To this end, we used the AAT to train HSAs, either to approach smiling faces or to avoid them. We examined whether such an AAT training could change HSAs' automatic avoidance tendencies, and if yes, whether AAT effects would generalize to a new approach task with new facial stimuli, and to mood and anxiety in a social threat situation (a video-recorded self-presentation). We found that HSAs trained to approach smiling faces did indeed approach female faces faster after the training than HSAs trained to avoid smiling faces. Moreover, approach-faces training reduced emotional vulnerability: it led to more positive mood and lower anxiety after the self-presentation than avoid-faces training. These results suggest that automatic approach-avoidance tendencies have a causal role in social anxiety, and that they can be modified by a simple computerized training. This may open new avenues in the therapy of social phobia.

Social anxiety disorder (SAD; American Psychiatric Association, 2000) is a common and debilitating disorder, associated with social and occupational impairment and considerable comorbidity with other psychiatric disorders (Stein and Kean, 2000). In the absence of effective treatment, SAD usually runs a chronic and disabling course (Dewit et al., 1999).

A large body of research on SAD attempted to identify factors playing a role in the etiology and maintenance of the disorder (e.g., Hirsch and Clark, 2004). One maintaining factor is the avoidance of threatening stimuli, for instance, social interactions (Wong and Moulds, 2011). According to Turk et al. (2001), avoidance prevents effective processing of the situation and disconfirmation of negative beliefs. Avoidance can be controlled and available to self-inspection, such as safety behaviors like wearing make-up to hide blushing (Wells et al., 1995), or it can be automatic, like keeping more distance from others (Rinck et al., 2010), or avoiding eye contact when looking at faces (Moukheiber et al., 2010). According to Voncken et al. (2011), such subtle avoidance behavior is especially relevant in the maintenance of SAD, as it might deteriorate the quality of interactions, which in turn may elicit more negative evaluations by others.

Since avoidance is partly automatic, indirect measures are needed for its assessment. A technique to assess implicit avoidance behavior is the Approach-Avoidance Task (AAT; Rinck and Becker, 2007). The AAT is based on the finding that pleasant stimuli elicit automatic approach tendencies, whereas unpleasant or threatening ones produce automatic avoidance tendencies (Chen and Bargh, 1999). Translating this into overt behavior (arm movements), approach is associated with pulling objects closer and avoidance with pushing them away (e.g., Rinck and Becker, 2007). In the AAT, participants see single pictures presented on a computer screen. On each trial, they move a joystick to make the picture disappear. When the joystick is pushed, the picture shrinks, when it is pulled, the picture grows in size. This correspondence of movement and visual feedback creates a strong impression of pulling the picture closer (approach) vs. pushing it away (avoidance). Typically, response times are correlated with picture valence: pleasant pictures are pulled closer more quickly, whereas unpleasant pictures are pushed away more quickly.

Heuer, Rinck and Becker (2007) used the AAT for studying approach-avoidance tendencies in social anxiety. They found automatic avoidance of smiling and angry faces in socially anxious participants (HSAs), reflected by shorter reaction times for pushing than for pulling. While the avoidance of angry faces may be considered adaptive and useful, automatic avoidance of positive social cues such as smiling faces is specific to HSAs. Notably, the bias was found although HSAs evaluated smiling faces positively in an explicit rating task. These findings of automatic avoidance of smiling faces in social anxiety were replicated by Lange et al. (2008) and Roelofs et al. (2010). According to Heuer et al. (2007), this implicit avoidance tendency might play a critical role in the maintenance of SAD, as it could interrupt adequate behavior in social interactions and increase anxiety caused by interactions.

However, the existing studies do not allow us to conclude that automatic approach-avoidance tendencies do indeed play a causal role in SAD. The observed avoidance of smiling faces might just as well be a symptom rather than a cause of SAD. In order to establish causal relations, an experimental manipulation of approach-avoidance tendencies is needed. Therefore, the current study attempted to train HSAs either to approach or to avoid smiling faces, using the same AAT that has earlier been used to measure smile-avoidance tendencies. Hence, the main questions of the current study were whether (1) such training would be effective in changing approach-avoidance tendencies in HSAs, and if so, (2) whether the effects would generalize to a new approach-avoidance situation with new faces, and (3) whether the training would affect subjective fear in a stressful social situation.

First, we expected that HSAs in the approach-smiling-faces training group would display a reduction in their tendency to avoid smiling faces after the training (i.e., be faster in pulling them closer). For HSAs in the avoid-smiling-faces training group, we expected that their tendency to avoid smiling faces would increase from pre- to post-assessment. Second, we hypothesized that the training effects would carry over to new faces in a new type of Approach-Avoidance Task, the Face-Turn AAT (FT-AAT; Voncken et al., 2011). Finally, we expected that HSAs in the approach-smiling-faces group would rate their mood more positively and would show less anxiety than HSAs in the avoid-smiling-faces group after giving a video-recorded self-presentation. Our expectations were based on previous findings showing that the modification of cognitive processes such as attention, associations, or approach-avoidance tendencies may have beneficial effects on disorders such as social phobia (Beard and Amir, 2008) or generalized anxiety disorder (Amir et al., 2009), or prevent relapse in treated alcoholics (Wiers et al., 2011; Eberl et al., 2013). Indeed, Taylor and Amir (2012) recently showed that a training to approach smiling and neutral faces did increase socially anxious participants' social approach behavior in a subsequent social interaction situation. However, in this study, no avoid-smiling faces condition was employed, and no effects of the training on state anxiety were found. Therefore, these results are encouraging, but they do not tell us whether approach-avoidance of positive social cues, i.e., smiling faces, has the postulated causal effects on anxiety in social situations.

## Methods

### Participants and design

Forty undergraduate students of Radboud University Nijmegen who scored high on a social anxiety pre-screening participated in this study in return for course credits. Of those, eight participants were excluded from further analyses because their scores on the Social Interaction Anxiety Scale (SIAS; Mattick and Clarke, 1998) were not elevated at the time of testing^1^. Participants were randomly assigned to one of two training conditions (approach-smiling-faces-and-avoid-checkerboards or vice versa), yielding 16 participants in each training group. The two groups did not differ on level of social anxiety or demographic variables (see Table 1). AAT reaction times (before and after the training), FT-AAT reaction times, and mood ratings before and after a social threat task were used as the dependent variables.

**Table 1:** **Means and standard deviations of demographics, questionnaire scores, AAT effects in ms, mood ratings, and anxiety ratings**.

**Training**	**Approach-Smile *n* = 16**	**Avoid-Smile *n* = 16**
% Female	69%	81%
Age	20.7 (2.1)	20.8 (2.7)
LSAS-Fear	30.8 (11.1)	34.7 (8.6)
SIAS	37.1 (10.5)	37.3 (8.9)
SDS	40.6 (8.0)	38.5 (9.0)
AAT-pre	11 (105)	−63 (171)
AAT-post	120 (197)	−97 (156)
Mood rating 1 (Pre training)	5.3 (4.5)	6.5 (5.3)
Mood rating 2 (Post training)	2.9 (3.6)	5.0 (5.2)
Mood rating 3 (Post face-turn AAT)	4.0 (3.1)	5.8 (5.2)
Mood rating 4 (Pre self-presentation)	−0.56 (5.0)	1.6 (5.9)
Mood rating 5 (Post self-presentation)	4.9 (5.1)	2.3 (6.3)
Anxiety rating 1	1.1 (1.2)	1.3 (1.7)
Anxiety rating 2	0.69 (0.87)	0.81 (1.3)
Anxiety rating 3	0.44 (0.73)	0.94 (1.4)
Anxiety rating 4	2.3 (1.6)	2.3 (1.6)
Anxiety rating 5	1.1 (1.5)	2.3 (1.7)

### Materials and procedure

#### Questionnaires

Participants first gave informed consent, then filled out the fear sub-scale of the Liebowitz Social Anxiety Scale (LSAS; Liebowitz, 1987), the (SIAS; Mattick and Clarke, 1998), and the Zung Self-Rating Depression Scale (SDS; Zung, 1965). Those scoring high on the SDS would be excluded from further analyses, although this was not necessary as all participants scored at or below average.

#### Video rating

Subsequently, participants watched a 1-min video in which a woman, supposedly a previous participant, described herself (e.g., her hobbies, interests, etc.). Thereafter, participants were asked to rate this person according to 4 different aspects, namely: “How attractive/friendly/sympathetic/competent does this person seem?”. This part was incorporated to make the final part of the experiment (see below) more plausible.

#### Mood ratings

After the video-rating, participants were asked to rate their mood by evaluating 3 negative statements (“How anxious/nervous/bored are you at the moment?”) and 3 positive ones (“How happy/comfortable/relaxed are you at the moment?”) on a 7-point scale (0= *not at all*, 6= *very much*). These mood ratings were repeated after each task during the experiment to assess changes in mood.

#### Pull-push AAT

Afterwards, the Pull-Push AAT followed. All participants were instructed to categorize pictures according to their *color* (gray vs. sepia) as quickly as possible, using a joystick. They always pulled the joystick toward themselves in response to gray pictures, and pushed it away in response to sepia pictures. The joystick (a Logitech Attack 3) was positioned about halfway between participant and computer, tightly fastened to the table. The stimuli were (a) smiling male and female faces derived from the Karolinska Directed Emotional Faces (KDEF; Lundqvist et al., 1998), and (b) neutral checkerboards. Each trial of the AAT was started by moving the joystick to the middle position and pressing the “fire button” of the joystick. Then a single picture was presented in medium size on the computer screen. A zoom function was employed, such that the picture grew in size when the joystick was pulled, and it shrank when it was pushed. The picture disappeared only when the joystick was moved completely into the correct direction. Response latencies were recorded automatically as the difference between time of picture appearance and disappearance.

Unbeknown to the participants, after 10 practice trials, the first part of the task was a pre-assessment phase (40 trials), in which they pulled and pushed both smiling faces and checkerboards (10 trials for each of the 4 combinations) to measure their pre-existing behavior tendencies. Without any obvious change or interruption, the pre-assessment phase changed into a training phase of 480 trials (240 faces, 240 checkerboards). For participants assigned to the *approach-smiling-faces group*, smiling faces were always gray-colored (approach), and checkerboards always sepia-colored (avoidance) in this phase. In the *avoid-smiling-faces group*, checkerboards were always gray (approach) and smiling faces always sepia (avoidance). After 440 training trials, and again without any obvious change in procedure, a post-assessment phase was inserted, which was identical to the pre-assessment phase. A comparison of the post-assessment to the pre-assessment was used to verify whether participants learned the intended approach-avoidance reactions. The task ended with the remaining 40 training trials. Subsequently, participants executed a dot-probe task^2^ which lasted for approx. 10 min.

#### Face-turn AAT

Afterwards, participants performed a Face-Turn Approach-Avoidance Task (FT-AAT), as described by Voncken et al. (2011), to measure whether the training effects generalized to a different approach-avoidance situation with new stimuli. Here, we used pictures of the faces of slightly friendly looking individuals (half male, half female) and pictures of computer monitors. In this task, joystick movements did not cause changes in picture size, but they made the depicted individuals or monitors turn toward the participant vs. away from him/her. At the beginning of each trial, the heads of the individuals would face to the left or to the right, and similarly, the front of the monitors would be directed to the left or to the right. Participants were instructed to push away all left-directed stimuli and to pull closer all right-directed ones. When pulling the joystick, faces (or monitors) turned around toward the participant in steps of 30 degrees, such that the frontal view of the face (or monitor) became apparent at the end (approach). Pushing away faces (or monitors) resulted in turning away from the participant with the back of the head (or monitor) as end point (avoidance). This task consisted of 12 practice trials and 96 experimental trials. The latter involved 16 pull trials and 16 push trials each for male faces, female faces, and monitors.

#### Social stress task

After this task, participants were asked to give a video-taped, one-minute self-presentation, comparable to the previously rated video. Participants video-recorded the presentation themselves, using a Logitech QuickCam. They were told that their video would be shown to the next participant, and that the next participant would evaluate the self-presentation in the way the current participant had just evaluated the previous participant. All participants agreed to this procedure, and no one doubted its validity. Nevertheless, to protect the participants' privacy, no video was actually presented to anybody else, and all videos were deleted after data collection was finished.

Participants also rated their mood both after the instructions (assessing fearful expectation) and after actually giving the self-presentation (assessing stress recovery). Comparisons of the two training groups at these two measurements served as our main dependent variable. Finally, participants gave some demographic information and completed an awareness check on paper. The overall experiment lasted about 50 min.

## Results

### AAT: manipulation check

To test our first hypothesis that approach-avoidance tendencies could be trained, two new dependent variables were computed from the participants' median AAT reaction times (RTs), indicating their face-approach tendency relative to their checkerboard-approach tendency. One variable contained this information for the pre-assessment, and the other for the post-assessment. The mean values of these scores are shown in Table 1, positive values indicate a relative approach tendency for smiling faces, negative ones a relative avoidance tendency for them. As expected, the groups' approach-avoidance tendencies did not differ significantly from each other before training, *t*_(30)_= 1.48, n.s. After the training, they did differ in the expected direction, *t*_(30)_ = 3.45, *p* = 0.002, with participants of the approach-faces training showing a significant face-approach tendency (+120 ms), *t*_(15)_ = 2.43, *p* = 0.03, and participants of the avoid-faces group showing a significant face-avoidance tendency (−97 ms), *t*_(15)_= 2.48, *p* = 0.03. Thus, the Pull-Push AAT yielded the expected training effects.

### Face-turn AAT

To test the hypothesis that AAT training effects generalize to a new AAT, a 2 (Training: approach-smiling-faces-avoid-checkerboards vs. vice versa) × 2 (Stimulus type: face, monitor) × 2 (Movement type: approach vs. avoidance) repeated-measures ANOVA was conducted on the median Face-Turn AAT reaction times. This analysis did not reveal the expected three-way interaction, *F*_(1, 30)_ < 1, n.s. However, closer inspection of the means suggested a difference between male and female faces, therefore three exploratory 2 (Training: approach-smiling-faces-avoid-checkerboards vs. vice versa) × 2 (Movement type: approach vs. avoidance) repeated-measures ANOVAs were added, one for each picture type. The analysis for *female* faces yielded the expected two-way interaction between Movement type and Training, *F*_(1, 30)_= 8.16, *p* < 0.01, η^2^_*p*_ = 0.22, but it was not found for male faces or monitors, both *F* < 2, *ns*. Further analyses of the female faces revealed that participants who had been trained to approach smiling faces did indeed approach female faces faster than participants who had been trained to avoid the faces, *t*_(30)_ = 2.24, *p* = 0.04 (see Table 2 for means and standard deviations).

**Table 2:** **Face-Turn-Approach-Avoidance Task: mean RTs and standard deviations in ms**.

**Training**	**Stimulus type**	**Movement type**	
		**Approach**	**Avoidance**
Approach-smile	Female	703 (137)	749 (177)
	Male	734 (159)	702 (173)
	Monitor	711 (133)	684 (154)
Avoid-smile	Female	800 (106)	735 (127)
	Male	786 (139)	750 (125)
	Monitor	785 (148)	704 (93)

### Mood ratings

To test our hypothesis that the approach-smiling-faces group would rate their mood more positively, an overall mood score was created for each of the 5 mood-rating phases (M1–M5). This was done by subtracting the sum score of all negative mood items (anxious, nervous, bored) from the sum score of all positive mood items (happy, comfortable, relaxed). As such, a positive value of this overall mood score indicates a positive mood state, whereas a negative value indicates a negative mood state^3^. As we were particularly interested in the participants' mood directly before and after the self-presentation, only the corresponding M4 and M5 scores were entered into the analyses (however, all scores are reported in Table 1). As a first step, we conducted a repeated-measures ANCOVA on the overall mood scores, including the between-subjects factor Training (approach-smiling-faces-avoid-checkerboards vs. vice versa) and the within-subjects factor Time (M4 vs. M5). Mood scores at M1 were added as a covariate to control for pre-experimental mood differences. This analysis revealed the expected Training × Time interaction, *F*_(1, 29)_ = 8.1, *p* < 0.01, η^2^_*p*_ = 0.22. When investigating this interaction further, results of paired-samples *t-tests* demonstrated that for participants of the approach-faces training, mood after the self-presentation was significantly better than directly before, *t*_(15)_ = 4.39, *p* < 0.01. No significant difference was found for participants of the avoid-faces training, *t*_(15)_ = 0.52, *ns*.

In addition, we executed two ANCOVAs of the overall mood scores at M4 and M5 separately (again making use of mood scores at M1 as covariate). There was no significant mood difference between the two training groups at M4 before the social threat task, *F*_(1, 29)_ = 0.73, *ns*. Afterwards at M5, however, participants of the approach-faces training group reported a significantly more positive mood than those of the avoid-faces training group, *F*_(1, 29)_ = 4.68, *p* = 0.04, η^2^_*p*_ = 0.14. See Table 1 for means and standard deviations of these scores.

### Anxiety ratings

Since the training was designed to specifically affect social anxiety, we also investigated its effects on the participants' anxiety. For this analysis, we used only one of the six mood ratings, namely the “How anxious are you at the moment?” rating. Again, we focused on the ratings directly before and after the self-presentation, computing the same analyses as above. The repeated-measures ANCOVA of the anxiety ratings yielded the expected Training x Time interaction, *F*_(1, 29)_ = 7.52, *p* = 0.02, η^2^_*p*_ = 0.21. Participants of the approach-faces training were less anxious after the self-presentation than before, *t*_(15)_ = 4.84, *p* < 0.001. In contrast, participants of the avoid-faces training reported a comparable level of anxiety before and after the self-presentation, *t*_(15)_ = 0, *ns*. Correspondingly, the two groups did not differ regarding their level of anxiety before the self-presentation, *F*_(1, 29)_ = 0.03, *ns*. Afterwards, the approach-faces group reported less anxiety than the avoid-faces group, *F*_(1, 29)_ = 5.05, *p* = 0.04, η^2^_*p*_ =0.15. Please see means and standard deviations of these scores in Table 1.

## Discussion

The main goals of this study were to examine (a) whether a Pull-Push-AAT (Heuer et al., 2007) could serve as an appropriate method to influence automatic approach-avoidance tendencies in response to smiling faces, and if so, (b) whether the training effects would generalize to a new approach-avoidance situation with new faces, and (c) whether the training would affect subjective anxiety in a real social threat situation.

Our findings confirmed our first hypothesis that the AAT-training is suitable for changing HSAs' avoidance of smiling faces. Specifically, HSAs trained to approach smiling faces became faster in pulling smiling faces closer to themselves. After the training, they showed a significant face-approach tendency. Similarly, participants trained to avoid smiling faces showed a significant face-avoidance tendency. Hence, we can argue that the AAT is an appropriate and promising method to train HSAs to approach smiling faces. Moreover, it may have potential therapeutic value in the sense that it could help to reduce *automatic* avoidance behavior in social anxiety, in addition to the existing therapeutic tools for reducing *controlled* avoidance behavior.

Furthermore, our findings partially confirmed our second hypothesis as well. The trained action tendencies of the Pull-Push AAT seemed to generalize, to some extent, to new faces in a new type of AAT, the Face-Turn-AAT (Voncken et al., 2011). Here, HSAs trained to pull smiling faces closer were faster to turn female faces toward themselves. However, it must be noted that no effect on male faces was observed. An explanation for this finding could be that the female faces were perceived as less threatening by the predominantly female HSAs of the current experiment. Accordingly, future studies should determine if and how the training effects can be extended to male faces in order to increase the effectiveness of the training for treatment purposes. These studies should also pay attention to possible interactions of participant gender, model gender, and emotion because there is evidence that the processing of smiling facial expressions depends on the gender of both the person expressing them and the person perceiving them (LaFrance et al., 2003).

Finally, we expected that when experiencing a social challenge, HSAs who had received approach-smiling-faces training would feel generally better and would experience less anxiety than HSAs who received avoid-smiling-faces training. The reported mood ratings and anxiety ratings were in accordance with this hypothesis. Compared to HSAs of the avoid-faces training, HSAs of the approach-faces training reported both higher positive mood in general, and lower levels of anxiety in particular, after videotaping their self-presentation and expecting it to be evaluated by a peer. Interestingly, there were no differences between the two groups in general mood or specific anxiety when they received instructions for the self-presentation. This suggests that the training affected mood recovery after stress rather than anticipatory fear: HSAs who had been trained to approach smiling faces appeared to *recover* more easily after the social threat task. This result is in accordance with studies that tested the effects of another type of training, attention bias modification, on emotional vulnerability (MacLeod et al., 2002; Amir et al., 2008). As in our study, only participants' anxiety *after* the stressful event was affected by the training condition. Moreover, as in many previous studies (e.g., MacLeod et al., 2002), our training was not a selective mood induction: directly afterwards, both training groups felt slightly worse than before. This is understandable, given the somewhat boring nature of the training.

In the current study, we experimentally manipulated whether the participants were trained to approach or to avoid smiling faces. This way, we can safely conclude that automatic approach-avoidance tendencies are indeed causal factors in the behavior and subjective fear of socially anxious individuals. This is important from a theoretical point of view, e.g., for the evaluation of cognitive theories of anxiety. However, it does not yet prove the therapeutic value of an approach training in SAD because the positive approach-smiling faces training was not compared to a placebo training condition. In this respect, our study nicely complements the one recently reported by Taylor and Amir (2012) who found positive effects of an approach-faces training, compared to a placebo training condition. In this study, however, the authors only found effects of the training on social interaction behavior, not on subjective fear. For the latter, several differences between the two studies may be responsible. For instance, our social stress task may have been more threatening, or our training may have been more powerful because it involved the approach of smiling faces rather than neutral faces. Therefore, future studies should compare the approach-smiling faces training to a placebo training condition, preferably in diagnosed social phobics, and study effects on both behavior and social anxiety levels. Moreover, studies with delayed follow-up measurements would be helpful for determining the duration of approach-avoidance training effects.

Several limitations of the current study deserve mentioning. First, the sample was rather small, and it contained more female than male participants, therefore it was impossible to test the participant gender × model gender × emotion interaction mentioned above. Also, we had neither a non-anxious control group nor a group of SAD patients to compare the pre-training AAT scores to. Moreover, the study is lacking emotional expressions other than happy faces. Finally, the exact mechanisms by which the current training reaches its positive effects on stress recovery remain to be determined. We have reason to assume that more than a response bias was induced, given the observed partial transfer to another task and the effects on mood. At least two mechanisms might be at work here. First, operant evaluative conditioning: approaching certain stimuli repeatedly can make these stimuli more pleasant, compared to repeatedly avoiding them (e.g., Woud et al., 2011). This effect seems to be strongest for ambivalent stimuli, which smiling faces are for HSAs. Second, if the smiling faces were experienced as threatening, their repeated presentation might have caused habituation and extinction of the fear reaction, and this effect might have been stronger for those participants who approached the faces without experiencing any negative consequences.

To summarize, we have demonstrated that it is possible to change avoidance tendencies in HSAs using an AAT training procedure, and that the training effects partly generalize to other situations involving automatic approach behavior. Moreover, training HSAs to approach rather than to avoid smiling faces led to less self-reported anxiety after a threatening social task, thereby fostering recovery from stress. These results corroborate the causal role of automatic approach-avoidance tendencies in social anxiety. Moreover, the results suggests that the AAT is not only suitable for the assessment of biased avoidance behavior in social anxiety, but also for the re-training of this automatic behavior. Since avoidance behavior plays an important role in the maintenance of SAD, an effective training to approach smiling faces could be an important step toward the improvement of social interactions in socially anxious individuals. Thus, the approach-smiling-faces training could be a promising addition to more traditional treatments of social anxiety.

### Conflict of interest statement

The authors declare that the research was conducted in the absence of any commercial or financial relationships that could be construed as a potential conflict of interest.

## Footnotes

^1^These 8 participants had SIAS scores lower than 21. Additional analyses including them yielded very similar results, except that the observed effects were smaller.

^2^The dot-probe task was intended to measure effects of the approach-avoidance training on attention bias, but due to a design error, its results could not be interpreted. Therefore, it is not described in detail here. Information about it can be obtained from the first author.

^3^We also analyzed the sum score of the positive mood items and the sum score of the negative items separately. This yielded results which mirrored those of the joint analysis reported here.
